# A cross-sectional study of hepatitis C among people living with HIV in Cambodia: Prevalence, risk factors, and potential for targeted screening

**DOI:** 10.1371/journal.pone.0183530

**Published:** 2017-08-23

**Authors:** Anja De Weggheleire, Sokkab An, Irith De Baetselier, Pisith Soeung, Huy Keath, Veasna So, Sreyphors Ros, Syna Teav, Bart Smekens, Jozefien Buyze, Eric Florence, Johan van Griensven, Sopheak Thai, Sven Francque, Lutgarde Lynen

**Affiliations:** 1 Department of Clinical Sciences, Institute of Tropical Medicine Antwerp, Antwerp, Belgium; 2 Infectious Diseases, Sihanouk Hospital Center of Hope, Phnom Penh, Cambodia; 3 Gastroenterology Hepatology, University of Antwerp, Antwerp, Belgium; 4 Laboratory of Experimental Medicine and Paediatrics, University of Antwerp, Antwerp, Belgium; Centers for Disease Control and Prevention, UNITED STATES

## Abstract

The epidemiology of hepatitis C in Cambodia is not well-known. We evaluated the prevalence of hepatitis C virus (HCV) and risk factors in the HIV cohort of Sihanouk Hospital Center of Hope in Phnom Penh to strengthen the evidence for suitable HCV testing strategies among people living with HIV (PLWH) in Cambodia. All consenting adult PLWH without a history of HCV treatment were tested for HCV between November 2014 and May 2016 according to the CDC algorithm (HCV antibody II electro-chemiluminescence immunoassay, followed by COBAS^®^ AmpliPrep/COBAS^®^ TaqMan^®^ HCV PCR and INNO-LIA^®^ HCV Score immunoblot end-testing). Genotyping was performed using the line probe assay Versant HCV genotype 2.0^®^. The study enrolled a total of 3045 patients (43% males, median age: 42.5 years, <1% high-risk). HCV antibodies were detected in 230 (7.6%; 95% confidence interval [CI] 6.6–8.5). Upon further testing, HCV antibodies were confirmed in 157 (5.2%; 95% CI 4.4–6.0) and active HCV in 106 (3.5%; 95% CI 2.8–4.2). Viremic prevalence peaked among men aged 50–55 years (7.3%) and women aged >55 years (11.2%). Genotype 1b (45%) and 6 (41%) were predominant. Coinfected patients had a higher aspartate-to-platelet ratio index, lower platelets, a lower HBsAg positivity rate and more frequent diabetes. Based on logistic regression, blood transfusion antecedents (adjusted odds ratio 2.9; 95% CI 1.7–4.9), unsafe medical injections (2.0; 1.3–3.2), and partner (3.4; 1.5–7.6) or household member (2.4; 1.3–3.2) with liver disease were independently associated with HCV in women. However, having a tattoo/scarification (1.9; 1.1–3.4) and household member (3.1; 1.3–7.3) with liver disease were associated with HCV in men. Thus, our study found intermediate endemicity of active hepatitis C in a large Cambodian HIV cohort and provides initial arguments for targeted HCV screening (>50 years, partner/household member with liver disease, diabetes, increased aspartate-to-platelet ratio index) as efficient way forward.

## Introduction

Hepatitis C virus (HCV) is a major global health problem. Approximately 1.1% of the world population, 80 million people, is chronically infected and HCV-attributable mortality accounted for 495,000 deaths in 2015 [1,2]. People living with HIV (PLWH) are disproportionately affected; prevalence is globally higher in this subpopulation but varies widely with the mode of transmission and type of exposure [3,4]. A recent systematic review revealed a HCV seroprevalence of 4% in heterosexually infected PLWH and 82% among PLWH who inject drugs. The latter group accounted for 58% of HCV/HIV coinfections globally [5].

In many countries, especially resource-constrained countries such as Cambodia, the extent and character of the HCV/HIV co-epidemic is poorly documented. The low success rate of pegylated interferon and ribavirin treatment, the only option until mid-2015, discouraged HCV testing in HIV programs despite its recommendation by the World Health Organization (WHO) since 2006. Now, with the increasing availability and affordability of highly efficacious and well-tolerated HCV direct-acting antiviral (DAA) treatments, more accurate mapping of the co-epidemic has become urgent for health program planning.

In Cambodia, most of the 70,000 adult PLWH are on antiretroviral treatment (>75% coverage) but unaware of their HCV status. According to national program data, PLWH in Cambodia have predominantly been exposed to HIV heterosexually and rarely through high-risk sexual behavior (2.1%) or injection drug use (0.5%) [6,7]. The prevalence of HCV/HIV coinfection is poorly documented. The available data are rather divergent (5.3% and 10.5% seroprevalence) but higher than the regional median of 3% for heterosexually exposed PLWH [5,8,9]. Information is even more scant for the prevalence of active HCV coinfection, as this requires HCV RNA testing; only Lerolle et al provided an estimate of 6.1% [9].

The particularly high medical injection and infusion usage rates in Cambodia, in combination with poor infection control until the late 1990s, are thought to have been the main drivers of the current HCV (co)epidemic in Cambodia and explain a higher overall HCV prevalence than the surrounding countries [10–17]. A recent local outbreak of HIV re-emphasized that unsafe medical injection practices still continue, though on a different scale [18]. Specific studies on risk factors for HCV infection in Cambodia are few and small in size, but supportive of this iatrogenic hypothesis; household and behavioral factors have not been found to be associated [19,20].

In the current study, we aimed to further the knowledge on the burden and character of the HCV/HIV coinfection epidemic in Cambodia by documenting age- and gender-specific prevalence, as well as risk factors for HCV infection in one of Cambodia’s largest HIV cohorts.

## Materials and methods

### Study design, population, and setting

We conducted a cross-sectional study in the outpatient HIV clinic of Sihanouk Hospital Center of Hope (SHCH) in Phnom Penh (Cambodia) between November 2014 and May 2016. Free-of-charge HIV care is offered in this non-governmental clinic as part of the national program. In November 2014, 3285 patients on antiretroviral therapy (ART) and 123 patients who were not on ART were in follow-up. The study population included all adult HIV patients in regular follow-up (minimum two consultations in the last 6 months). Patients with antecedents of HCV treatment were excluded. We targeted 3000 enrollments.

### General study procedures

Via a questionnaire on the day of blood sampling, data on socio-demographics (including sexual orientation, engagement in sex work), risk factors for HCV, and clinical history were collected. Questioned risk factors were: having a partner (husband/wife or sexual partner) with liver disease, having a household member (excluding husband/wife or sexual partner) with liver disease, sexual orientation, having a tattoo/scarifications, ever receiving a blood transfusion, ever undergoing surgery (including year), frequency of past (before enrollment in HIV care) and current injections and intravenous (IV) fluid use, purpose of injections (medical/recreational), and ever receiving injections/IV fluids by non-sterile/re-used/glass material. Data on past commercial sex activities were collected from the HIV patient’s file.

In addition to HCV diagnosis, laboratory analyses included hepatitis B surface antigen (HBsAg), full blood count (FBC), aspartate transaminase (AST), alanine transaminase (ALT), CD4, and HIV viral load (VL).

### Laboratory testing

Blood (16 mL) was collected through venipuncture. This amount allowed for both immediate testing at SHCH for HCV antibodies, HBsAg, FBC, ALT, and AST and dispatch to national reference laboratories for CD4 (National Institute of Public Health) and HIV VL (National Center for HIV/AIDS, Dermatology and STD). The remaining blood was stored and used for quantitative HCV-RNA testing (private laboratory Paramed, Phnom Penh), confirmatory HCV immunoblot (Institute of Tropical Medicine—ITM, Antwerp, Belgium) and/or external quality control (EQC) when indicated.

**Fig 1:**
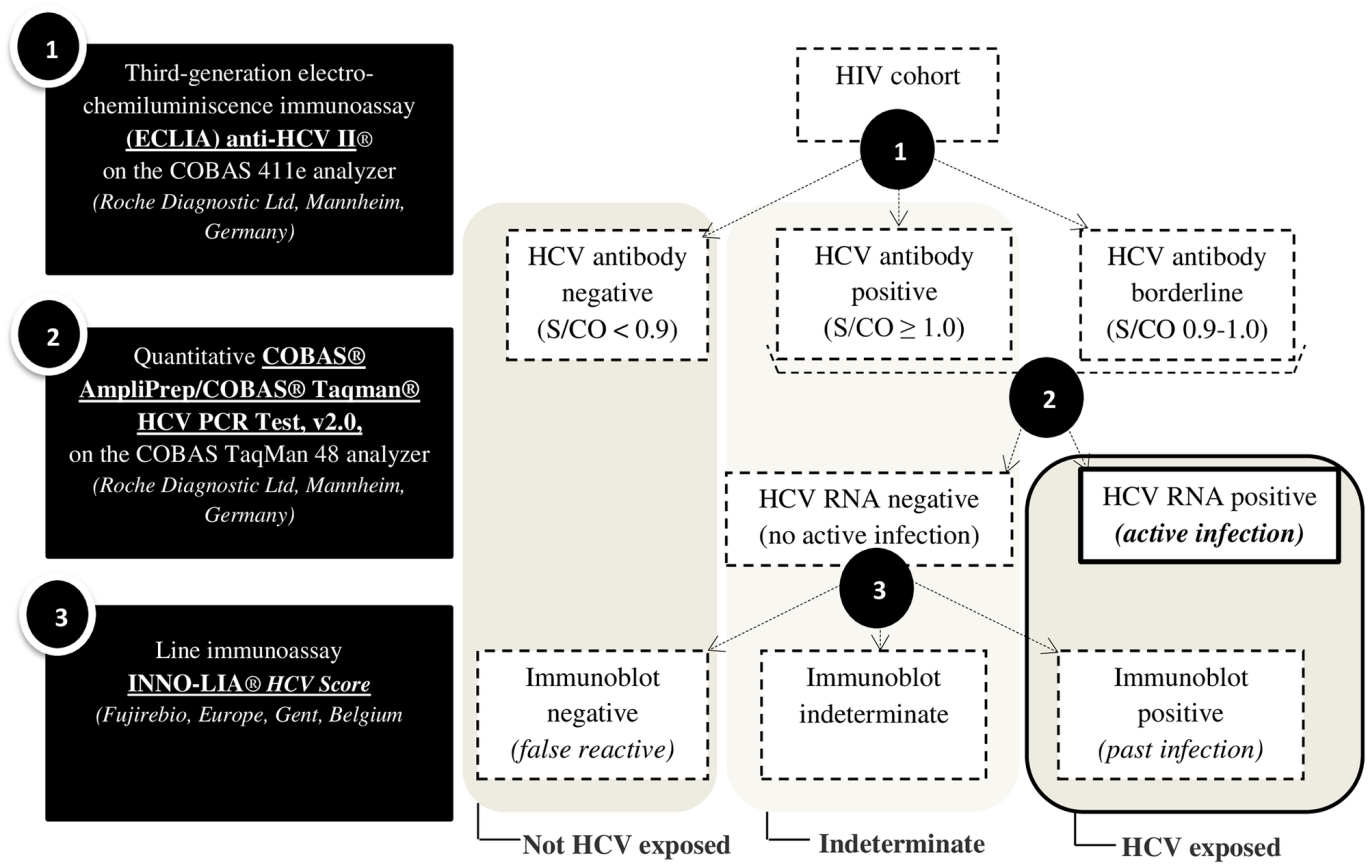
HCV testing algorithm, tests used, and prevalence measures.

Hepatitis C testing (Fig 1) followed the Center for Disease Control algorithm [21]. Initial testing for HCV antibodies was performed using the third-generation electro-chemiluminescence immunoassay (ECLIA) ELECSYS^®^ HCV antibody II (Roche Diagnostics Ltd, Mannheim, Germany). Samples with a signal-to-cutoff ratio (S/CO) <0.9 were considered non-reactive (negative), those with an S/CO between 0.9 and 1.0 borderline, and those with an S/CO ≥1.0 reactive (positive). All samples with an S/CO >0.9 were further tested for HCV-RNA using the quantitative COBAS^®^ AmpliPrep/COBAS^®^ TaqMan^®^ HCV PCR Test, v2.0, on the COBAS^®^ TaqMan^®^ 48 Analyzer (Roche Diagnostics Ltd, Mannheim, Germany). The lower limit of detection was 15 IU/mL. Finally, to differentiate biologically false reactive from resolved infections, samples with an S/CO ratio >0.9 but undetectable HCV-RNA were tested with the score line immunoassay INNO-LIA^®^ HCV (Fujirebio Europe). Band reactivity was graded positive, negative, or indeterminate for HCV antibodies by visual calibration against control bands according to the manufacturer’s instructions.

ALT and AST were determined on the Cobas Integra 800 analyzer (Roche Diagnostics Ltd., Mannheim, Germany). Normal values were ALT <41 IU/L and AST <40 IU/L for males, and ALT <33 IU/L and AST <32 IU/L for females. The FBC was measured on the Sysmex KX-21 (Sysmex Corporation, Kobe, Japan) and HBsAg on the Cobas 411e instrument using the HBsAg II assay (Roche Diagnostics).

Genotyping was performed using the reverse-hybridization line probe assay (LiPA), Versant HCV genotype 2.0 (Siemens Healthcare, NY, USA) at the Belgian National HCV Reference Center (consortium of the Institute of Public Health and Université Catholique de Louvain).

As EQC, we organized HCV antibody re-testing for 5% of the negative samples at ITM using the VITROS HCV antibody reagent on the VITROS 5600 (Ortho Clinical Diagnostics, Bridgend, UK), and quantitative HCV-RNA re-testing for 10% of the HCV-RNA positive samples and 10% of HCV-RNA negative samples at the University of Antwerp, Belgium (amplification by Abbott m2000 real-time PCR).

### Criteria for HCV infection status

Patients were categorized into five groups after HCV antibody (Ab) testing by ECLIA, confirmatory HCV-RNA PCR, and INNO-LIA immunoblot end-testing (Fig 1): group 1, active HCV infection (borderline/positive by ECLIA and detectable HCV-RNA); group2, past HCV infection (borderline/positive by ECLIA, undetectable HCV-RNA, and positive INNO-LIA); group 3, negative HCV Ab test (negative by ECLIA); group 4, false positive HCV Ab test (borderline/positive by ECLIA, but undetectable HCV-RNA and negative INNO-LIA); and group 5, indeterminate group (borderline/positive by ECLIA, undetectable HCV-RNA, and indeterminate INNO-LIA), as different explanations are possible (false reactivity, impaired antibody production, and recovery from a distant infection with remaining low immune response) [22,23]. Group 5 was excluded from the risk factor analysis. Groups 1 and 2 together were considered “confirmed HCV exposed”. Groups 3 and 4 together were considered “confirmed not HCV exposed”. In group 4, sero-reversion after recovery of a distant infection is also possible, but this is considered rare and not considered as such in our analyses [22].

### Statistical analysis

Data were analyzed using Stata version 11.2 (StataCorp LP, College Station, TX, USA). Measures of prevalence were estimated, expressed in percentages, and reported with Wilson 95% confidence intervals (CIs). Descriptive statistics, using medians and interquartile ranges for continuous variables and percentages for categorical variables, were used to summarize characteristics of the enrolled cohort. Subgroups were compared by Pearson’s chi-squared test (or Fisher’s exact test) for categorical variables and the Wilcoxon rank sum test for continuous variables. P<0.05 was considered significant.

Logistic regression was used to estimate, with odds ratios (ORs) and 95% CIs, the association between the outcome variable (i.e., confirmed HCV exposed) and potential exposure factors (iatrogenic, partner, household member). Associations were initially investigated using bivariable analysis. Confounding and/or interaction by gender and age was examined by the Mantel-Haenszel method and test of homogeneity, and in further analysis by taking into account stratification (gender) and including age in the multivariable models. Initial selection of exposure factors for the multivariable model was based on the bivariable analysis (P<0.20) and a backward elimination approach to identify the final model retaining only those with P<0.05.

### Ethical considerations

Enrolled patients provided voluntary signed informed consent, or a fingerprint if illiterate, prior to inclusion. The study was approved by the Institutional Review Board of ITM Antwerp, the Ethics Committee of Antwerp University Hospital (Belgium), and the Cambodian National Ethics Committee for Health Research. The study was registered at clinical trials.gov (NCT02361541). HCV treatment provision was foreseen for the patients identified with HCV co-infection, starting with those in urgent need (advanced fibrosis).

## Results

### HIV cohort characteristics

**Fig 2:**
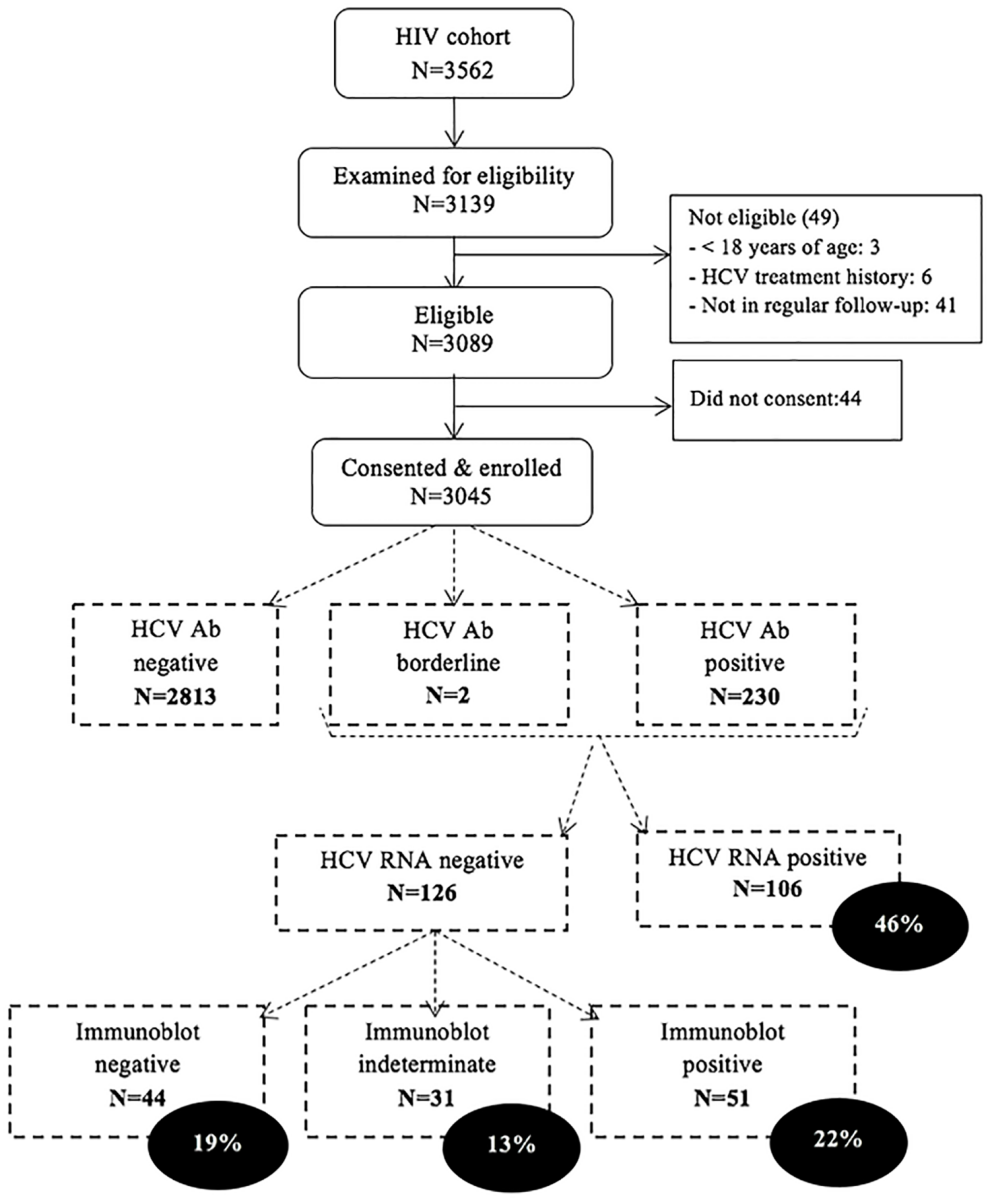
Flowchart of study enrollment and hepatitis C diagnostic results.

During the study, 3139 of 3562 potentially eligible adult PLWH were examined for eligibility; 3089 were eligible and 3045 consented to be enrolled in the study (Fig 2).

**Fig 3:**
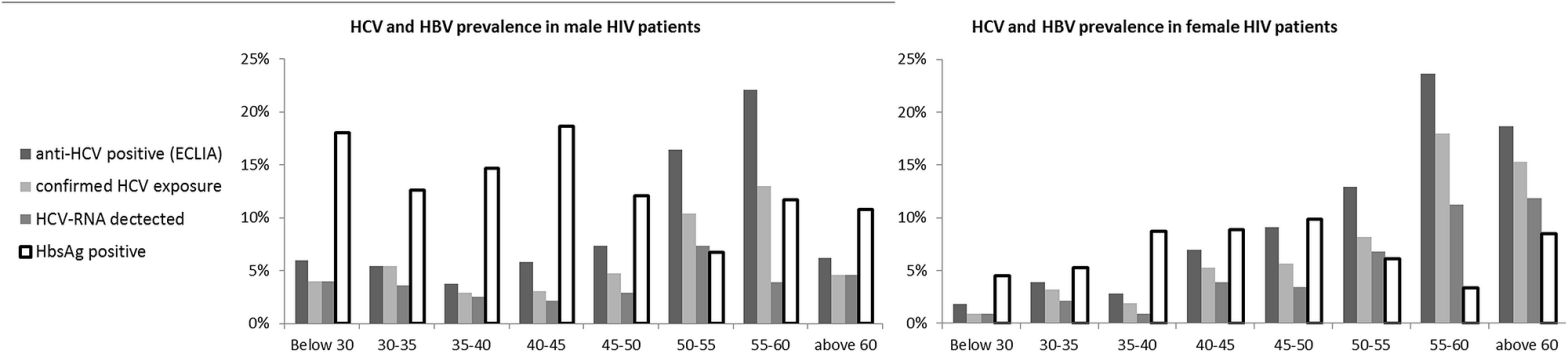
Age- and gender-specific prevalence of HCV and HBV. ECLIA = electro-chemiluminescence immunoassay. Confirmed HCV exposure = positive for HCV antibodies with ECLIA and positive for HCV-RNA or HCV INNO-LIA immunoblot.

The median age of enrolled patients was 42.5 years (interquartile range 36–48), male/female ratio 0.75 and duration of HIV follow-up at SHCH 7.3 years (Table 1). Most patients when starting HIV follow-up (92.7%) and upon enrollment in the study (87.7%) were residing in the capital Phnom Penh or one of the five surrounding provinces. The majority (97.6%) were on ART (duration: 6.9 years), with a median CD4 count of 464 cells/μL and 96.6% with an undetectable HIV VL (<50 copies/mL). Ninety-two percent were on a non-nucleoside reverse-transcriptase inhibitor (NNRTI)-based regimen. The overall HBsAg positivity rate was 10.2%, but it was significantly different between men (13.7%) and women (7.6%, P<0.001). The highest HBsAg prevalence was found in men <45 years of age (16.4%, Fig 3).

**Table 1:** Socio-demographic and clinical characteristics of the enrolled HIV patients according to current HCV infection status.

	All enrolled HIV patients N = 3045	HIV without active HCV N = 2939	HCV/HIV coinfected N = 106	P-value
Male	1307 (42.9)	1262 (42.9)	45 (42.5)	0.92
Age, years	42.5 (36.3–48.1)	42.3 (36.3–47.7)	48.2 (42.1–53.2)	**<0.001**
Body mass index: ≤18.5 kg/m^2^	435 (14.3)	421 (14.3)	14 (13.2)	0.74
**HIV care**				
Duration HIV care, years	7.3 (4.6–9.7)	7.3 (4.6–9.7)	6.6 (2.9–9.9)	0.09
CD4 nadir, cells/μL^†^	131 (37–251)	130 (37–250)	141 (45–261)	0.39
Receiving ART	2972 (97.6)	2869 (97.6)	103 (97.2)	0.74
Duration on ART, years	6.9 (4.4–9.1)	6.9 (4.4–9.1)	6.0 (3.1–9.0)	0.06
Switched ART for presumed ART-related hepatotoxicity*	150 (5.6)	137 (5.3)	13 (12.9)	**0.001**
**Current ART regimen**				
NNRTI or PI based:				
EFV	1539 (51.8)	1476 (51.5)	63 (61.2)	**0.01**
NVP	1189 (40.0)	1160 (40.4)	29 (28.2)	
PI	232 (7.8)	223 (7.8)	9 (8.7)	
AZT or TDF containing:				
TDF	1239 (41.7)	1183 (41.2)	56 (54.4)	**0.001**
AZT	1653 (55.6)	1613 (56.2)	40 (38.8)	
AZT and TDF	20 (0.7)	19 (0.7)	1 (1.0)	
**Laboratory results**				
HIV VL undetectable^‡^	2517 (96.6)	2437 (96.6)	80 (97.6)	1.00
HCV-RNA, ×10^3^ IU/mL^§^	NA	NA	2.000 (515–4.550)	
CD4, cells/μL	464 (339–609)	465 (340–611)	406 (310–558)	**0.04**
ALT, IU/L	28 (20–43)	28 (19–42)	47 (30–64)	**<0.001**
AST, IU/L	26 (21–36)	26 (21–35)	48 (32–70)	**<0.001**
Hemoglobin, g/dL	12.7 (11.6–13.9)	12.7 (11.6–13.9)	12.6 (11.7–13.6)	0.76
Platelets, ×10^9^ cells/L	266 (221–312)	267 (223–313)	210 (159–255)	**<0.001**
APRI	0.29 (0.21–0.41)	0.28 (0.21–0.40)	0.64 (0.43–1.18)	**<0.001**
**Symptoms/signs**				
Fatigue/weakness	142 (4.7)	132 (4.5)	10 (9.4)	**0.02**
Diffuse pruritus	120 (3.9)	110 (3.7)	10 (9.4)	**0.003**
Myalgia/arthralgia	154 (5.1)	140 (4.8)	14 (13.2)	**<0.001**
Anorexia/weight loss	110 (3.6)	102 (3.5)	8 (7.6)	**0.03**
Abdominal pain/nausea	93 (3.1)	89 (3.0)	4 (3.8)	0.56
Sensory neuropathy	96 (3.2)	91 (3.1)	5 (4.7)	0.39
**Current comorbidities/conditions**				
HBsAg positivity	311 (10.2)	309 (10.5)	2 (1.9)	**0.002**
Diabetes mellitus	113 (3.4)	100 (3.4)	13 (12.5)	**<0.001**
Arterial hypertension	451 (14.8)	429 (14.6)	22 (21.2)	0.07
Alcohol use >140 g/week	75 (2.5)	75 (2.6)	0 (0.0)	0.11
Tuberculosis	26 (0.9)	21 (0.7)	5 (4.7)	**0.002**
**Past comorbidities/conditions**				
Alcohol >140 g/week (before enrollment in HIV care)	343 (11.3)	331 (11.3)	12 (11.3)	0.99
Tuberculosis in the past (≥1 episode since enrollment in HIV care)	1130 (37.1)	1090 (37.1)	40 (37.4)	0.89

ART: antiretroviral therapy, EFV: efavirenz, NVP: nevirapine, PI: protease inhibitor, TDF: tenofovir, AZT: zidovudine, VL: viral load, APRI: AST-to-platelet ratio index, HBsAg: hepatitis B surface antigen, NA: not applicable. Data are presented as n (%) or median (interquartile range).

*Includes only hepatitis B surface antigen-negative patients. Missing values (if >10):

^†^CD4 baseline (129 missing values),

^‡^HIV VL (368 missing values),

^§^CD4 at enrollment (11 missing values)

Key populations were confirmed rare. Only 0.6% (N = 20) of the cohort identified as homosexual; 0.2% (N = 6) as having antecedents of injection drug use, none of current use; and 0.2% (N = 5) as having engaged in sex work in the past. Seventy-five patients (2.5%), mostly men, reported moderate to heavy alcohol intake (>140 g/week).

### Hepatitis C prevalence

Using the electro-chemiluminescence assay, 230 (7.6%) of the 3045 enrolled PLWH tested positive for HCV antibodies (Fig 2). Two had a borderline result. Upon confirmatory HCV-RNA testing in all ECLIA-positive and borderline samples, 106 (45.7%) tested positive. Immunoblot end-testing of the 126 HCV-RNA negative samples revealed resolved infection in 51 (22%), false reactivity in 44 (19%), and indeterminate results in 31 (13.4%).

In our cohort, 157 patients (5.2%; 95% CI 4.4–6.0) had confirmed HCV exposure and been effectively infected with HCV with or without subsequent natural clearance thereafter. Active hepatitis C was diagnosed in 106 (3.5%; 95% CI 2.9–4.2).

Despite the overall confirmed HCV exposure prevalence and viremia being similar among men and women (5.2% vs. 5.1% and 3.4% vs. 3.5%, respectively), important age-specific differences were found (Fig 3). The prevalence significantly increased with age. Patients >50 years of age were most affected (HCV exposure in 3.7% vs. 11.2%, P<0.001; viremia 2.5% vs. 7.5%, P<0.001). Among women aged over 55 years, more than 10% had an active HCV infection. Younger men (<40 years) were proportionally more affected than women of the same age (3% vs. 1.3%, P = 0.046).

The prevalence of active hepatitis C was not significantly different among PLWH residing in Phnom Penh (72/1905, 3.8%), the immediate surrounding Kandal province (17/387, 4.4%), the other five neighboring provinces (6/324, 1.9%), and the remaining provinces (11/429, 2.6%). Notably higher active HCV prevalence was found among PLWH from two riverine districts, Preaek Pnov (12/31, 38.7%) in Phnom Penh and Mukh Kampul (6/41, 14.6%) in Kandal. Coinfected patients from Preaek Pnov were younger (median age: 40.4 years).

### Characteristics of HCV/HIV coinfected patients

Patient characteristics according to coinfection status are summarized in Table 1. PLWH with active HCV were older (48.2 years, interquartile range 42–53) than the rest of the cohort. None of the coinfected reported sex work, being MSM, or person who injects drugs (PWID). The duration on ART was not significantly different for those with or without HCV coinfection (6.9 vs. 6 years, respectively). Excluding HBsAg-positives from the analysis, as all switches to improved hepatitis B active ART regimens were noted as a switch for hepatotoxicity, ART regimens were switched more frequently for presumed ART-related hepatotoxicity in HCV/HIV coinfected patients (12.5% vs 5.3%). HIV infection was generally well controlled in coinfected patients; 98% had an undetectable VL and the median CD4 count was >400 cells/μL. Tuberculosis (TB) was more frequent in coinfected patients at the time of enrollment in the study, but the proportion that had had one (or more) TB episode since enrollment in HIV care was not significantly different.

Median ALT and AST levels were significantly higher, whereas the platelet count was lower in the HCV coinfected patients. The median aspartate-to-platelet ratio index (APRI), which is commonly used as a surrogate biomarker for liver fibrosis, was also significantly higher in coinfected patients. Among all enrolled patients, 25 had a platelet count < 100×10^9^ cells/L; 20 of them had active viral hepatitis (9 HBsAg positive, 11 HCV-RNA positive).

Symptoms such as fatigue, diffuse pruritus, myalgia/arthralgia, and anorexia/weight loss were more frequent among HCV coinfected PLWH. Among the questioned comorbidities, only diabetes mellitus was more common in the coinfected (12.5% vs. 3.4%, P<0.001). The difference remained significant when adjusting for age (adjusted OR [aOR] 2.9, 95% CI 1.5–5.4). HBsAg positivity was rare in HCV/HIV coinfected patients (1.9% vs. 10.5% in the rest of the cohort; P = 0.002).

Genotype results were available for 87 patients. Most patients had genotype 1b (N = 39; 44.8%) or 6 (N = 36; 41.4%). Genotypes 1a (N = 7), 2 (N = 4), and 3 (N = 1) were less common. Genotyping with the Versant assay remained incomplete for 16 samples (impossible to differentiate between genotype 1 and 6), uninterpretable for one sample and HCV-RNA was too low for two samples.

### HCV exposure and risk factors

Table 2 summarizes the factors associated in unadjusted models with HCV exposure in the total population and stratified by gender. Among both men and women, being >50 years of age and having a household member with liver disease were significantly associated with positive HCV antibody status. For women, most of the other assessed potential iatrogenic risk factors, such as having been transfused, having undergone surgery before 2000/2005, and having received injections or infusions with non-sterile/re-used/glass syringes, were positively associated, as well as having a partner with liver disease. For men, having a tattoo or scarifications was the only factor other than age >50 years and a household member with liver disease that was found to be associated.

**Table 2:** Univariable and multivariable analyses of hepatitis C risk factors.

	HCV exposed N = 157	HCV non-exposed N = 2857	Crude odds ratio (95% CI)	P-value	Gender-stratified odds ratio (95% CI) with P-value				P-value	Adjusted odds ratios (95% CI) with P-value			
					Male		Female			Male		Female	
**Female**	89 (56.7)	1636 (57.3)	0.98 (0.71–1.35)	0.89									
**Aged > 50 years**	67 (42.7)	517 (18.1)	3.37 (2.42–4.69)	**<0.001**	2.86 (1.73–4.73)	**<0.001**	3.9 (2.48–6.09)	**<0.001**	0.37	**3.00 (1.81–4.97)**	**<0.001**	**3.43 (2.15–5.48)**	**<0.001**
**Tattoos, scarifications**	26 (16.6)	316 (11.1)	1.59 (1.03–2.46)	0.05	1.92 (1.09–3.36)	**0.02**	1.21 (0.57–2.56)	0.62	0.33	**1.90 (1.07–3.36)**	**0.03**		
*Missing*: *5*													
**Transfusion**	36 (22.9)	265 (9.3)	2.89 (1.95–4.29)	**<0.001**	1.78 (0.89–3.60)	0.10	3.84 (2.35–6.27)	**<0.001**	0.08			**2.91 (1.74–4.87)**	**<0.001**
*Missing*: *14*													
**Surgery**	44 (28.0)	691 (24.2)	1.22 (0.85–1.75)	0.28	0.84 (0.44–1.59)	0.59	1.51 (0.97–2.36)	0.07	0.14				
*Missing*: *1*													
**Surgery before 2000**	17 (10.8)	177 (6.2)	1.84 (1.09–3.11)	**0.03**	0.65 (020–2.11)	0.45	2.99 (1.63–5.50)	**<0.001**	0.02				
*Missing*: *1*													
**Surgery before 2005**	23 (14.7)	289 (10.1)	1.52 (0.96–2.41)	0.09	0.69 (0.27–1.75)	0.43	2.29 (1.33–3.94)	**0.002**	0.02				
*Missing*: *1*													
**Partner**	13 (8.3)	75 (2.6)	3.34 (1.81–6.15)	**0.001**	2.11 (0.62–7.15)	0.22	4.09 (1.99–8.41)	**<0.001**	0.35			**3.42 (1.53–7.63)**	**0.003**
*Missing*: *11*													
**Household**	16 (10.2)	106 (3.7)	2.94 (1.69–5.11)	**0.001**	2.74 (1.17–6.21)	**0.01**	3.11 (1.48–6.53)	**0.002**	0.82	**3.09 (1.31–7.28)**	**0.01**	**2.39 (1.29–3.23)**	**0.04**
*Missing*: *2*													
**Non-sterile injections**	63 (40.4)	734 (26.1)	1.91 (1.38–2.66)	**<0.001**	1.19 (0.71–1.99)	0.51	2.81 (1.81–4.35)	**<0.001**	0.01			**2.04 (1.28–3.23)**	**0.002**
*Missing*: *50*													
**Injections in last 3 months (≥ 1)**	12 (7.6)	240 (8.4)	0.90 (0.49–1.65)	0.74	0.92 (0.32–2.58)	0.87	0.90 (0.42–1.88)	0.78	0.98				
**Injections in past (more)**	26 (16.6)	510 (17.9)	0.91 (0.59–1.41)	0.68	1.11 (0.58–2.10)	0.76	0.79 (0.44–1.42)	0.43	0.45				

Data are presented as n (%) unless otherwise noted. We considered only those with confirmed HCV exposure status (N = 157) as HCV infected. Patients (N = 31) with indeterminate HCV antibody status (HCV antibody positive by ECLIA, undetectable HCV-RNA, and INNO-LIA indeterminate) were excluded from the analysis, as no conclusive arguments were found in their profile to categorize them confidently as false reactive or resolved from a distant HCV infection.

Age-adjusted multivariate models were constructed separately for men and women. In the final reduced model for women, having been transfused (aOR 2.9, 95% CI 1.7–4.9), having a partner (aOR 3.4, 95% CI 1.5–7.6) or household member (aOR 2.4, 95% CI 1.3–3.2) with liver disease, and having received medical injections with non-sterile material (aOR 2.0, 95% CI 1.3–3.2) remained independently associated with HCV exposure. For men, having a tattoo or scarifications (aOR 1.9, 95% CI 1.1–3.4) and a household member with liver disease (aOR 3.1, 95% CI 1.3–7.3) remained significantly associated in the age-adjusted model.

Importantly, 29% of HCV exposed patients did not report having been exposed to any of the above independent risk factors and were younger than 50 years of age.

## Discussion

In this study, 7.6% of the 3045 enrolled PLWH tested positive for HCV antibodies. Less than half (46%) had an active HCV infection. The seroprevalence is in the previously reported range for PLWH (5.3%-10.5%) and general population (2.3%-14.7%) in Cambodia [8,9,19,24,25]. The proportion with active infections was lower than expected. Data on comparable cohorts (low-risk profile, long-term on successful ART, predominance of interleukin-28B CC variant carriers, high proportion HCV genotype 6) are scarce. Lerolle et al reported that 62% of 52 HCV antibody-positive PLWH had active infection, but their patients were on ART for a shorter duration [9]. Spontaneous clearance after infection is more frequent in IL28B CC carriers [26], but whether HCV-RNA spontaneous clearance during the persistent infection phase, as a result of immune reconstitution following (long-term) ART, explains the higher proportion of HCV antibody positive/HCV-RNA negative patients merits further research. Such late spontaneous clearance has been documented on several occasions in IL28B CC carriers, and also more recently in CT carriers [27–29]. If and the extent to which it has contributed in our cohort cannot be answered by our data. In Siem Reap (Cambodia), among the general population, the proportion with active HCV was even lower (39.3%), which may indicate other underlying factors [19].

Overall, HCV prevalence did not differ among men and women in our study. However, gender and age-cohort differences were identified. Older age groups were more exposed to HCV, with the exception of men aged >60 years. Why this was the case remains unclear. Excess mortality in older exposed and chronically infected men could be a possible reason, as men more frequently have additional risk factors (e.g., alcohol use) for a more rapid progression to end-stage liver disease. Active coinfection was highest (above 10%) among women aged >55 years. Among the younger PLWH (<40 years), men remained at more substantial risk of HCV exposure than women. PLWH with overt hepatitis B (HBsAg positive) were very unlikely (0.6%) to be HCV co-infected. At this stage, we did not investigate whether and how viral interference with occult co-existence or excess mortality among triple-infected patients explains this phenomenon. In the context of increasing access to HCV interferon-free treatment and the risk of co-existing HBV reactivation during or after treatment, we recognize it would be worthwhile to further characterize the local extent of the hepatitis B and C co-epidemic (occult or not) [30,31]. Hepatitis B (HBsAg) was detected in 10.2% of the HIV patients in our study, which is similar to previously reported national and regional estimates [32,33]. Hepatitis B prevalence was significantly higher among men. The lower prevalence in older age groups can probably be explained by gradual inactivation of HBV replication and clearance, or excess mortality [34]. The prevalence of active HCV was high (11.5%) among PLWH with diabetes, which was expected [35], as was the considerably higher prevalence among PLWH of two neighboring (opposite riverbanks) districts in Phnom Penh and Kandal province. Whether these are indeed local epidemic hotspots merits further investigation (local survey, qualitative research, or pooling of existing data), as our numbers were small.

Key populations (PWID, sex workers, MSM) were rare in our study population similar to the overall national HIV cohort [6]. Therefore, for the HCV risk factor analysis, we focused on iatrogenic and household transmission. Blood transfusion, unsafe medical injections, and having a partner or household member with liver disease were independently associated with confirmed HCV exposure among women in our age-adjusted analysis. For men, only having a tattoo or scarifications and living with a household member with liver disease were independently associated. Though other studies in Cambodia or among Cambodian natives have already identified iatrogenic exposure as a risk factor, none have suggested a gender difference [19,20,36]. Our finding is coherent with the previously documented higher usage of injections/infusions among women (7.5 vs. 4.3 per person-year in men) [18] and the higher likelihood of exposure to potentially unsafe invasive medical procedures because of female healthcare needs (prenatal vaccination, abortion, delivery, anticonception). Our study also suggests that other routes of transmission (household and/or sexual, tattooing) should not be ignored. Tattooing is a common practice among men in Cambodia. Overall, 16% of men, and more than 20% of those <40 years of age in our cohort, have a tattoo. However, over-interpretation of our risk factor analysis should be avoided. We intended to explore, and were not exhaustive in the factors we assessed. In addition, some of the assessed factors include socially stigmatized and criminalized behaviors. As such, patients may have preferred not to disclose; thus underreporting of factors such as injection drug use, commercial sex activities, and homosexual identity is likely.

Other limitations of our study are its single-center approach which may raise questions about representativeness. PLWH not in care may have a different profile and be more likely to belong to vulnerable and stigmatized groups, such as PWID and MSM. As for those in care, national program data support our cohort being similar to the large majority of cohorts in care in Phnom Penh (~25,000 PLWH), with the exception of the two smaller clinics (~800 PLWH in follow-up) targeting most-at-risk populations [6]. The cross-sectional design limited the questions we could answer (e.g., clearance) and likely resulted in recall bias for distant risk factors. The presence of serosilent HCV was not assessed and, therefore, underestimation of HCV prevalence cannot be excluded, though this is expected to be a rare phenomenon in patients on long-term ART and with third-generation HCV antibody tests [37]. We did not note PLWH with a high degree of suspicion for HCV coinfection despite a negative HCV antibody result. The strengths of the study are the large number of included patients, compliance with good clinical and laboratory practices, and that it is the first purposely designed HCV prevalence study among PLWH in Cambodia.

Extrapolating from our findings, the number of HCV coinfected among adult PLWH currently in care in Cambodia may be between 1600 (low) and 2400 (high estimate). Therefore, ‘treatment for all’ seems to be a feasible objective, especially at current declining costs per treatment. However, the major difficulty and cost will lie in screening and diagnosis.

The WHO and other major HCV guidelines recommend that all PLWH should be screened for HIV based on the evidence of an overall higher prevalence in this subpopulation and the higher risk for faster evolution of HCV-associated liver disease [38–40]. Another reason is the ease of reaching PLWH who are in care. For the general population, the WHO recommends targeted approaches unless the prevalence is >2% or 5%. We think it can be questioned whether a ‘screen all PLWH without prioritization’ is the most commendable public health strategy at this initial stage of HCV screen and care scale-up in Cambodia, as resources (human resources, laboratory, and funding) are limited and the prevalence of low-to-intermediate level. It should be evaluated further whether, in the context of Cambodia, it would not be more efficient and feasible to tailor this recommendation and start also for PLWH with a targeted approach combining age and factors linked to disease severity [41]. Our data suggest that straightforward criteria, such as age >50 years or having a household member or partner with liver disease, could be used to identify priority groups for HCV screening among PLWH in Cambodia. Looking back on our study population, we could have identified 55% of the HCV/HIV currently coinfected by testing only 25% of the PLWH using this approach. Considering only age >50 years (20% of the cohort), 45% of cases would have been identified, matching a number needed to test (NNT) of 13. The NNT increases steeply in the younger age groups (40–50 years: NNT 32; <40 years: NNT 53). Other factors (e.g., presence diabetes, increased APRI) may also have potential for an easy-to-use clinical prediction tool to identify patients with active HCV infection in contexts in which universal screening is not feasible immediately, or to assure prioritization for those most likely to be infected in the setting of low-to-intermediate prevalence [42].

## Conclusion

We found low to intermediate levels of endemicity of active HCV infection in PLWH aged <50 years, but high levels among the older PLWH. The most plausible explanation is a cohort effect of patients infected many years ago. Therefore, birth-cohort testing may be the first and easiest targeted HCV screening strategy the national HIV program and its partners could consider applying if resources are limited. In addition, or for the other age cohorts, strategies triggered by an increased APRI score could be explored further.
